# Immune mediated pediatric encephalitis – need for comprehensive evaluation and consensus guidelines

**DOI:** 10.1186/s12883-020-1605-y

**Published:** 2020-02-03

**Authors:** Julia Shekunov, Caren J. Blacker, Jennifer L. Vande Voort, Jan-Mendelt Tillema, Paul E. Croarkin, Magdalena Romanowicz

**Affiliations:** 1 Department of Psychiatry and Psychology, Mayo Clinic, Rochester, MN USA; 2 Department of Psychiatry and Psychology, Mayo Clinic Health Systems, Austin, MN USA; 3 Department of Neurology, Mayo Clinic, Rochester, MN USA

**Keywords:** Encephalitis, Autoimmune, Neuropsychiatric, Psychiatric, Seronegative

## Abstract

**Background:**

Autoimmune encephalitis is characterized by neuropsychiatric symptoms associated with brain inflammation. The differential is usually broad and Psychiatry often collaborates with Neurology in diagnostic clarification and symptom management. At least 40% of neuroencephalitis cases are of unknown etiology which adds to difficulties in making the right diagnosis and deciding on the appropriate treatment (Granerod et al., Lancet Infect Dis 10:835-44, 2010). The aim of this case series was to present four cases with complicated psychiatric symptomatology and isolated neurologic signs and symptoms, evaluated at a large tertiary medical center and treated for suspected autoimmune encephalitis, demonstrating the complexity of diagnosis and treatment.

**Case presentation:**

Four diagnostically challenging and heterogeneous cases displayed clinical symptomatology suggestive of autoimmune encephalitis. All cases presented with neurologic and psychiatric symptoms, but had negative autoantibody panels, normal or inconclusive magnetic resonance imaging results and non-specific cerebrospinal fluid changes. All were challenged with immunosuppressive/immunomodulatory treatments with overall poor response rates.

**Conclusions:**

There is a heterogeneous presentation of autoimmune encephalitis in pediatric populations. In the absence of positive findings on testing, individuals who do not meet proposed criteria for seronegative encephalitis may be misdiagnosed, and/or may not respond adequately to treatment. In those cases, comprehensive evaluation and stringent application of consensus guidelines is necessary.

## Background

Autoimmune encephalitis is characterized by neuropsychiatric symptoms associated with brain inflammation. Multiple etiologies include autoantibodies to cell proteins, intracellular antigens, and paraneoplastic processes [1–3]. The estimated incidence of autoimmune encephalitis is 0.8/100,000/year, and prevalence 13.7/100,000 in children and adults [4]. Etiology of encephalitis varies depending on different geographic regions with majority of cases remaining unexplained [5]. Less is known about rates of pediatric autoimmune encephalitis.

Symptoms of autoimmune encephalitis can include cognitive regression/impairment, memory changes, seizures, sleep disturbance, autonomic instability, speech changes or mutism, and involuntary movements [6]. Psychiatric symptoms, including anxiety, agitation, delusions, and hallucinations can occur early in the course of autoimmune encephalitis [3]. There is commonly a subacute decline over the course of days to weeks, but symptoms can rapidly fluctuate, or present insidiously [7]. Children may be less likely to have severe autonomic manifestations [8], and are more likely to have neurologic manifestations than psychiatric [3, 9].

The differential is broad and Psychiatry is often consulted to assist with diagnostic clarification. Evaluation typically includes identifying the presence of clinical symptoms, evaluating biological abnormalities in serologic testing, and assessing paraclinical abnormalities via neuroimaging, electroencephalography (EEG), and lumbar puncture [2]. Antibody testing takes several days, response to immunotherapy can be slow, and over half of suspected autoimmune encephalitis cases are seronegative [10]. Accordingly, a clinical diagnostic approach has been developed, combining neurologic assessment, neuroimaging, and cerebrospinal fluid (CSF) testing, with levels of evidence established for possible, probable, or definite diagnoses of autoimmune encephalitis to support initiation of prompt immunotherapy where appropriate. Proposed diagnostic criteria for autoantibody-negative but probable autoimmune encephalitis include presence of rapid progression (less than 3 months) of working memory deficits, altered mental status or psychiatric symptoms; exclusion of well-defined syndromes of autoimmune encephalitis; reasonable exclusion of alternative causes; absence of well characterized autoantibodies in serum and CSF, and at least two of: magnetic resonance imaging (MRI) abnormalities suggestive of autoimmune encephalitis; CSF pleocytosis, CSF-specific oligoclonal bands or elevated CSF immunoglobulin G (IgG) index or both; or brain biopsy showing inflammatory infiltrates and excluding other disorders [11]. Pediatric criteria for diagnosis do not yet exist. Still, according to a recent systematic review [5], in many cases the causative agent of encephalitis remains unknown, despite advances in laboratory and imaging technology. This might suggest that there are a number of unknown pathogens that have not been associated with encephalitis and/or that some of the immune-mediated mechanisms are not well understood.

Treatment for autoimmune encephalitis is often empiric, and may involve corticosteroids, plasmapheresis and/or intravenous immunoglobulin (IVIG) [3, 12]. Rituximab is more often used in children due to its relatively favorable safety profile, as compared to cyclophosphamide [3]. Based on a retrospective study, etiology of acute encephalitis (that included encephalitis of unknown origin) is not associated with clinical treatment outcomes [13]. Treatment failures may be indicative of neurologic insult from past inflammation or ongoing inflammatory disease [14]. However, treatments are not without side effects, and therefore it is important not to expose patients unnecessarily. Clinical guidelines exist which direct clinicians through appropriate diagnostic and treatment decision trees [11].

**Fig. 1:**
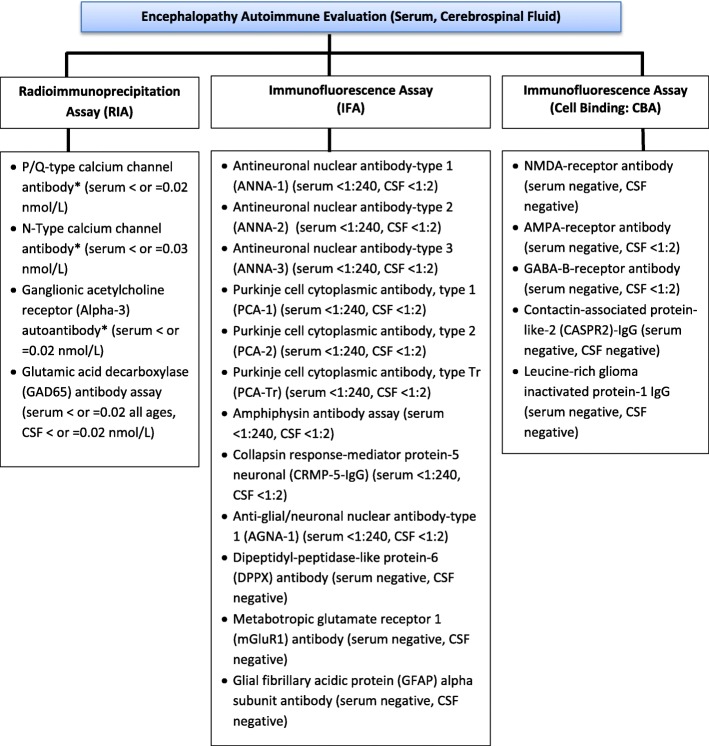
Summary of autoantibody testing in serum and spinal fluid in Mayo Clinic’s autoimmune encephalopathy panels with reference values in brackets. Test details and references obtained from Mayo Clinic Laboratory Test Catalog, https://www.mayocliniclabs.com/testcatalog/Overview/92116. (*Denotes testing completed in serum but not in spinal fluid)

We present four cases of children and adolescents with complicated psychiatric and neurologic symptomatology, evaluated at a large tertiary medical center, in whom autoimmune encephalitis was suspected and treatment was initiated. These cases were diagnostically challenging, with negative autoantibody panels, normal or inconclusive MRI results, non-specific CSF changes, and no tissue testing (either via immunochemistry in brain tissue or a neuronal cell culture). All received immunosuppressive and/or immunomodulatory treatments for autoimmune encephalitis based solely on clinical symptomatology. Table 1 summarizes each case, specific testing and outcome. Figure 1 describes the laboratory-specific autoantibody testing performed in serum and spinal fluid at our institution. Additional file 1 provides a method description of Mayo Clinic laboratory testing for autoimmune encephalopathy- evaluation, for cerebrospinal fluid and serum samples. The cases demonstrate both a need for better understanding of pediatric autoantibody-negative encephalitis, and also the importance of applying clinical guidelines to diagnosis and treatment, especially in cases where the diagnosis is not clear.

**Table 1:** Summary of physical, laboratory, imaging findings, treatment and outcomes for 4 cases of suspected autoimmune encephalitis. Clinical features suggestive of autoimmune encephalitis are bolded

Case	1	2	3	4
Age in years/sex	13/M	17/F	9/F	17/M
Symptom duration	8 months	3 months	1 year	6 months
Physical symptoms	**Anorexia** **Motor slowing** **Slow, ataxic gait** **Abnormal movements of lip/tongue** **Mutism** **Food refusal** **Dizziness** **Autonomic instability**	**Headaches** Abdominal pain Emesis Urinary retention Vibratory tactile sensation in head Dysphagia Constipation	**Periods of confusion** **Disorientation** **Decreased speech fluency** **Language regression Nonsensical speech**	**Decreased movements** **Decreased speech** **Soft, scripted speech** **Staring spells** **Fatigue**
Psychiatric symptoms	**Social isolation** **Catatonia**	**Memory difficulties** **Decline in academic performance** **Social isolation** **Anxiety** **Panic attacks** **Agitation** **Insomnia** **Paranoia**	**Inappropriate laughter** **Talking to imaginary friends** **Disengagement in school** **Aggression** **Defiance** **Social Isolation** **Visual hallucinations** **Paranoia** **Disorganized behavior**	**Decreased attention and concentration** **Social isolation** **Not caring for self** **Auditory and visual hallucinations** **Talking to self** **Irritability** **Difficulties with multistep commands** **Increased sleep**
Family History of autoimmune disease	None	None	Father with multiple sclerosis Paternal aunt with Myasthenia Gravis	None
Serum and urine	Mildly elevated GAD65 antibody (0.15 nmol/L), otherwise unremarkable including rest of encephalopathy panel, electrolytes including calcium (with exception of low phosphorus), folate, B12, Lyme serology, herpes simplex, enterovirus, cryptococcus, VDRL, whole exome sequencing	Mildly elevated GAD65 antibody (0.15 nmol/L), otherwise unremarkable including CBC, electrolytes, thyroid function, liver function, C-reactive protein, B12, ceruloplasmin, toxicology, heavy metals, blood smear, urinalysis	Unremarkable including outside hospital testing CBC, CMP, inflammatory markers, thyroid studies, ammonia, folate, copper, ceruloplasmin, heavy metals, plasma amino acids, urine organic acids, very long chain fatty acids, lysosomal disorders screen, chromosomal microarray, Noonan panel. Repeat inpatient testing unremarkable	Unremarkable except for low ferritin (12 mcg/L), including toxicology screen, CBC, electrolytes, inflammatory markers, thyroid function
MRI	Unremarkable other than evidence of malnutrition	Slit third ventricle with narrowed lateral ventricles, no cerebral hypotension and diffuse changes consistent with perinatal insult	Unremarkable	Unremarkable
CSF	Mildly elevated protein (45 mg/dL), otherwise unremarkable; no presence of bands	Total protein elevated (144 mg/dL; 110 mg/dL 6 months later); no presence of bands	Unremarkable; no presence of bands	Positive GFAP antibodies from outside hospital, repeat negative; no presence of bands
EEG	Unremarkable	Unremarkable	Intermittent diffuse nonspecific bifrontal slowing, bifrontal spikes and sharp waves without clinical correlate, intermittent independent left/right temporal slowing	Mild nonspecific background slowing
Treatment with IVIG or corticosteroids	5 days IV methylprednisolone; 5 days IVIG then monthly infusions for 3 months	5 days IVIG then intermittent doses; 5 days methylprednisolone upon readmission	3 days high dose IV methylprednisolone then oral prednisone; single dose IVIG then twice-weekly IVIG, then monthly IVIG; rituximab	5 days IV methylprednisolone
Response	Improved over time	Initial significant improvement, not sustained with both treatment course. Return to baseline after neurosurgical intervention	Some initial improvement in mood, speech, social interactions; not sustained	Initial improvement, not sustained

## Case presentation

### Case 1

A 13-year-old, developmentally normal boy, with no psychiatric history, was hospitalized for 8 months of progressive malnutrition, physical weakness, and mutism. He had been physically well until developing influenza followed by an observed tick bite without rash. Over the next 4 months, he developed abdominal pain with a single episode of bloody emesis. Abdominal computed tomography (CT) demonstrated mesenteric adenitis, a nonspecific finding; gastrointestinal biopsies were negative. Anorexia and motor slowing developed. By month six, gait became slow and shuffling, with abnormal lip/tongue movements. MRI-brain was unremarkable. He developed postural dizziness, falls, ataxic gait, and gradual mutism. Food refusal led to 30 pound weight loss, and severe malnutrition resulted in hospitalization.

During hospitalization, serum testing and sleep-wake EEG were unremarkable except serum was positive (15 nmol/L) for GAD65 antibody, a nonspecific finding. CSF was normal (GAD65 0.00), except for mildly elevated protein. Repeat MRI-brain showed evidence of malnutrition but was essentially normal. Neurological examination revealed bradykinesia, masked facies, oral dyskinesias, bilateral cogwheeling at wrists, knees, and elbows, brisk reflexes with clonus, absent Babinski bilaterally, positive Hoffman bilaterally, head tremor, mutism, and no volitional movements. Autonomic instability occurred: tachycardia and normothermic diaphoresis. A positive score of 14 for catatonia on a Bush-Francis rating scale [15], resulted in a benzodiazepine challenge up to 1 mg three times daily ultimately stopped for somnolence. Neurology recommended levodopa for extrapyramidal symptoms; 1 g intravenous (IV) methylprednisolone for 5 days and five total doses of IVIG for possible autoimmune encephalitis in the context of progressive weakness and difficulties initiating movement. Treatment was well tolerated and followed by motor symptom improvement over 1 to 2 weeks, including resolution of cogwheeling and rigidity, which did not recur upon discontinuation of levodopa, and resolution of tachycardia. At the time of transfer to inpatient rehabilitative therapy, the patient had improved oral intake, resolution of cogwheeling and rigidity, and more spontaneous movements, but was nonambulatory, unable to complete activities of daily living (ADLs), and essentially nonverbal.

In addition to suspected autoimmune encephalitis, there was suspicion for a functional communication disorder due to inconsistencies in his physical exam. Neither diagnosis alone could fully explain the patient’s presentation, and accordingly, he also received physical therapy, speech therapy and occupational therapy.

He was discharged home eating appropriately, ambulating, able to complete all ADLs, and mouthing words after a 50 day hospitalization for 3 months of monthly IVIG. At the time of the last follow-up he continued to have some difficulty with eating and used primarily gesturing, writing and facial expressions to communicate, though this was improving.

### Case 2

A 17-year-old female, with no psychiatric diagnoses, and a history of premature birth (24-week gestation), perinatal intraventricular hemorrhage, hydrocephalus, cerebral palsy, and ventriculoperitoneal shunt was hospitalized following 3 months of progressive mental status changes. She had no foreign travel or infectious contacts. Four months prior, she sustained a minor head injury without loss of consciousness. One month later, she began struggling with memory and homework. Her A/B grades declined and she did not complete the semester. She described vibratory tactile sensations in her head, severe headaches over occiput and vertex, and developed social isolation, anxiety, and panic attacks. New-onset insomnia was alleviated by zolpidem and lorazepam. One month prior to admission, she reported dysphagia, abdominal pain, constipation, urinary retention, vomiting.

At admission, physical exam was unremarkable besides moderate cognitive impairment (Kokmen 23/38) [16] and urinary retention requiring catheterization. Serum and EEG testing was unremarkable except a nonspecific finding of mildly elevated GAD65. CSF was remarkable for elevated protein of 144 mg/dL and CSF autoantibodies were positive for CASPR2- autoantibodies, though repeated testing was negative. MRI-spine was negative for cord tethering. CT-head demonstrated slit ventricles consistent with excessive shunting but thought to not adequately explain the patient’s subacute constellation of fatigue, memory, cognitive, and behavioral problems. MRI-brain showed slit third ventricle with narrowed lateral ventricles and diffuse changes consistent with perinatal insult. Psychiatry evaluated severe insomnia and behavioral changes. There was no evidence of a mood or anxiety disorder, or catatonia. Neuropsychological testing reflected low-average general cognitive functioning with weakness in nonverbal/visuospatial processing, consistent with complications of prematurity. Baseline cognitive testing was unavailable.

A 5 day course of 1 g per day IV methylprednisolone and IVIG for suspected autoimmune encephalitis were initiated. Lorazepam was provided on an as-needed basis for anxiety and insomnia secondary to use of methylprednisolone. Treatment was tolerated and within days, the patient began to walk normally, interact appropriately, and sleep better. She was discharged home with an IVIG taper. She required rehospitalization 6 months later for mental status changes and sleep disturbances. Repeat MRI-brain, EEG, CSF/serum testing were significant only for elevated CSF protein of 110 mg/dL. Five days of methylprednisone briefly resolved symptoms without sustained improvement. Her clinical care transferred to another facility where her over-draining shunt was replaced with a programmable shunt and she returned to baseline suggesting that shunt malfunction may contribute to psychiatric symptoms such as anxiety, confusion and memory loss. Patient was told that adults should not use pressure valves in their shunts and instead a magnetic adjustable valve is preferred option.

### Case 3

A nine-year-old girl, no psychiatric history, was seen for a second opinion of cognitive, behavioral, and speech/language regressions. She had been healthy until 11 months prior, with onset of inappropriate laughter, talking to imaginary friends, decreased speech fluency, and vocabulary loss. Teachers noted worsening social engagement and academic performance, disorientation, aggression, defiance, repetitive hand movements, and eloping from school. She described visual hallucinations and paranoia. Language became nonsensical. She became lost in familiar places and struggled to get home from the school bus.

The patient was evaluated at an outside facility and prescribed risperidone for suspected psychosis. EEG and MRI-brain were unremarkable. In month five, she was hospitalized for medical evaluation due to ongoing symptoms and concern for possible autoimmune encephalitis. Serum, CSF and urine studies were normal, as were repeat MRI-brain and CT-abdomen/pelvis. Repeat EEG showed nonspecific anterior slowing. The patient began empiric treatment for autoimmune encephalopathy, with IV methylprednisolone for 3 days and one dose of IVIG, then transitioned to oral prednisone. Steroids impaired sleep, increased appetite, and she became agitated/aggressive. She transferred to a rehabilitation facility where she had noticeable improvements in mood, clarity and fluency of speech, reading comprehension, and social engagement. As prednisone was tapered, she experienced symptom recurrence. The patient continued twice-weekly IVIG, tapered to once-monthly infusions, without sustained improvement.

At the time of presentation to our outpatient clinic, 11 months after symptom onset, she had intermittent visual hallucinations, disorganized thinking, impaired speech fluency, inappropriate laughter, pressured speech, and vague descriptions of an entity inside her. Risperidone was replaced with aripiprazole, with no meaningful improvement. Rituximab was started with slight improvement after two doses, though not continued after parents sought a second opinion due to perceived lack of response. Laboratory testing of serum and CSF was normal. EEG showed intermittent diffuse nonspecific 5 hertz slowing bifrontally, bifrontal spikes and sharp waves without clinical correlate, intermittent independent bitemporal slowing consistent with mild encephalopathy. Neuropsychological testing revealed borderline general intellectual functioning, and impaired adaptive functioning. Cognitive skills had regressed across time) but academic skills remained average, consistent with premorbid functioning. Ultimately, it was felt that the patient’s presentation reflected encephalopathy given the continued regression, involvement of fine motor skills, and reduced working memory/processing speed.

### Case 4

A 17-year-old autistic boy, previously taking aripiprazole between ages 12 and 13 for mood symptoms, was seen for a second opinion regarding mental status changes beginning 6 months prior. The patient attended school with 1:1 teacher-to-student ratio, and had several friendships. Following a vacation, and without evidence for prodromal illness, he struggled with attention and concentration, became withdrawn, moved less, stopped self-cares, and talked to himself. He described visual and auditory hallucinations. He displayed frequent staring spells but 24-h EEG showed no epileptiform discharges. He was hospitalized at an outside hospital with normal laboratory testing including MRI-brain and CSF studies except positive for serum glial fibrillary acidic protein (GFAP) antibodies (specific result unavailable). Testicular teratoma was ruled out by scrotal ultrasound. He was treated for suspected autoimmune encephalitis with IV methylprednisolone, 1 g per day for 5 days, with tolerability and initial significant improvement that subsequently slowed.

At the time of our assessment, parents reported irritability, auditory and visual hallucinations, quiet and impaired speech, difficulties following multi-step commands, and hypersomnolence. Psychiatry started aripiprazole for hallucinations. Overnight EEG showed non-specific mild background slowing. Repeat laboratory testing was normal except for low ferritin. Serum and CSF encephalopathy testing, MRI-brain and MRI-cervical spine were negative including negative GFAP antibodies. Creatine urine studies were slightly abnormal and were recommended to be repeated locally. With negative antibody testing, supportive care continued as parents noted ongoing gradual improvement.

## Discussion and conclusions

These cases were complex, necessitating multidisciplinary collaboration. All received treatment for autoimmune encephalitis based on clinical symptoms. All had different degrees of response to treatment, and diagnostic certainty was never satisfactorily achieved. These cases highlight how essential it is to use internationally agreed criteria for diagnosis and initiation of treatment of pediatric cases of suspected autoimmune encephalitis. Though proposed criteria for diagnosis exist [11], they are not consistently used. Additionally, even after acute treatment is completed, patients are frequently left with residual psychiatric symptoms that require ongoing management [17–19]. This may include pharmacologic and psychotherapeutic treatment of anxiety, panic, obsessive-compulsive disorder, irritability, inattention, impulsivity, depression and psychosis [2].

Many cases of autoimmune encephalitis are characterized by rapid symptom progression that include memory issues, movement disorders, seizures, insomnia, issues with speech, significant behavior changes, psychosis, and obsessive–compulsive like symptoms [20]. Outcomes may vary from full recovery to death [21]. Time to recovery differs across patients, with some evidence suggesting that more than 50% of patients with Hashimoto’s encephalopathy improve in the first month of treatment, yet other types of encephalitis show poorer treatment response and longer time to recovery [3, 20, 22]. All four patients in this series were treated for autoimmune encephalitis yet had limited or unsustained response to short courses of first-line treatments of IVIG and steroids. The only patient who ultimately returned to baseline also had a premorbid neurosurgical condition. A second-line treatment (rituximab) was employed only in Case 3, though not continued by parents. There is increasing literature on other treatment options including other second-line therapies like cyclophosphamide, mycophenolate mofetil and azathioprine, and novel medications such as IL-6 blockade and proteasome inhibitors for refractory cases [12, 14, 20, 23].

Autoimmune encephalitis often involves psychiatric symptoms, movement disorders, memory and speech issues, fluctuating consciousness, and autonomic instability [24, 25]. Cases 1/3/4 demonstrated four of these clinical characteristics. All cases displayed psychiatric symptoms. There were speech issues in Cases 1/3/4, memory difficulties in Cases 2/3/4, and movement changes in Cases 1 and 2. Communication and/or behavioral difficulties prevented standardized memory and cognitive testing for all cases. Autonomic instability was present only in Case 1, who had symptoms consistent with catatonia. Diagnosis of autoimmune catatonia is challenging particularly with negative autoimmune testing [26].

Though clinically suggestive of autoimmune encephalitis, assessment and diagnosis of our cases was complicated by lack of specific biologic or paraclinical abnormalities. All were seronegative, with no MRI or CSF abnormalities. Non-specific EEG changes were reported in Cases 3/4. None received tissue-based testing. These cases were treated for autoimmune encephalitis yet did not meet Graus criteria for probable or definite autoimmune encephalitis [13]. This may be a contributing factor in the limited response to immunosuppressive and immunomodulatory treatments observed in these patients.

Lack of diagnostic clarity also raises questions about whether treatment in these cases was justified. Their presentations were acute, severe and their clinical symptomatology was suggestive of autoimmune encephalitis but they did not meet current consensus guidelines for this diagnosis. Despite improvements in diagnostic technology, there are still a significant number of cases of unknown etiology. The diagnostic conundrum of cases presented here is that they all displayed psychiatric symptoms, but at the time their presentation could not be explained by psychiatric diagnosis alone. Use of clinical guidelines, applied in a systematic way, might have been helpful in the care of these patients. For example, although negative autoantibodies do not preclude definitive diagnosis of autoimmune encephalitis, they can be useful for determining subtype, treatment choice, and prognosis [11]. Additionally, when managing an autoantibody negative case, further testing should be considered as per the consensus guidelines, and this should always include CSF if serum is negative. Confirmatory tests should be strongly considered, such as cell-based assay and tissue immunohistochemistry [11]. Testing for autoimmune encephalitis is evolving and changing rapidly. Laboratory tests for new antibodies are being discovered and experts in the field recognize that there is still much to learn about diagnosis of autoimmune encephalitis. This poses difficulty in being able to properly diagnose. On the other hand, treatments used for autoimmune encephalitis are not without side effects and potential complications. Weighing the risks and benefits of treatment requires an evaluation of the patient’s functional impairment, side-effects [27], the reasonable safety profile of IVIG [28] and other treatment options, and the risk of progressive decline in the absence of treatment. Improved outcomes have been associated with immunotherapy, early initiation of treatment, and use of second and third-line treatments if necessary [29]. This often poses a clinical dilemma: how aggressive should the treatment be in cases where diagnosis is unclear? In the adult population, the consensus seems to be that aggressive treatment should be considered in most situations due to a high likelihood of favorable outcomes [13]. The data is lacking in the pediatric population and no official guidelines are present. However in light of potential side-effects of immune treatment, autoimmune encephalitis criteria is pragmatically made more stringent when no biomarker or inflammation can be found as in cases of encephalitis of unknown etiology. By contrast, if a known and clinically relevant antibody is identified, the same criteria may be relaxed to provide the necessary treatment for the patient. We specifically chose the cases for this series to outline diagnostic and treatment challenges in the field and to illustrate that ultimately the treatment of these individuals is based on the empirical evidence accrued to the best of ability and is far from definitive at the present time.

There is a heterogeneous presentation of neuropsychiatric features of autoimmune encephalitis in pediatric population. Since patients with autoimmune encephalitis often present with physical and psychiatric symptomatology, psychiatrists and neurologists are both commonly involved in their care. Cases with negative autoantibody panels, normal/inconclusive MRI results, and non-specific CSF changes, but with clinical symptomatology suggestive for autoimmune encephalitis are difficult to manage and not definitive when no biomarker can be identified. There is a need for comprehensive evaluation with use of consensus guidelines for diagnosing seronegative autoimmune encephalitis.

## Supplementary information

**Additional file 1.** Method description of Mayo Clinic laboratory testing for Encephalopathy-Autoimmune Evaluation, cerebrospinal fluid and serum samples.

## Data Availability

Not applicable.

## Abbreviations

ADLs: Activities of daily living
AMPA: α-amino-3-hydroxy-5-methyl-4-isoxazolepropionic acid
CASPR2: Contactin Associated Protein 2
CBC: Complete blood count
CMP: Complete metabolic panel
CSF: Cerebrospinal fluid
CT: Computed tomography
EEG: Electroencephalography
GABA-B: Gamma-aminobutyric acid B
GAD65: Glutamic acid decarboxylase 65
GFAP: Glial fibrillary acidic protein
IgG: Immunoglobulin G
IV: Intravenous
IVIG: Intravenous immunoglobulin
LGI1: Leucine-rich, glioma inactivated 1
MRI: Magnetic resonance imaging
NMDA: N-methyl-D-aspartate
VDRL: Venereal Disease Research Laboratory test
