# Supplementary material for: Immune mediated pediatric encephalitis – need for comprehensive evaluation and consensus guidelines
Source: BMC Neurol. 2020 Feb 3;20:44. doi: 10.1186/s12883-020-1605-y (PMC6996166; doi:10.1186/s12883-020-1605-y)
Supplement: Supplementary file 1 — Additional file 1. Method description of Mayo Clinic laboratory testing for Encephalopathy-Autoimmune Evaluation, cerebrospinal fluid and serum samples. [file 12883_2020_1605_MOESM1_ESM.docx]

**Supplementary 1.** Method description of Mayo Clinic laboratory testing for Encephalopathy-Autoimmune Evaluation, cerebrospinal fluid and serum samples.

**CEREBROSPINAL FLUID**

**Indirect Immunofluorescence Assay (IFA):** Before testing, patient's specimen is pre-diluted. After applying to a composite substrate of frozen mouse tissues (brain, kidney, and gut) and washing, fluorescein-conjugated goat-antihuman IgG is applied to detect the distribution and pattern of patient IgG binding.(Pittock SJ, Kryzer TJ, Lennon VA: Paraneoplastic antibodies coexist and predict cancer, not neurological syndrome. Ann Neurol 2004;56:715-719)

**Radioimmunoassay (IPA):** (125)I-labeled recombinant human GAD65 and nonimmune human serum are incubated with the patient's diluted CSF. Antihuman IgG and IgM are then added to form an immunoprecipitate. After washing the precipitated immune complexes, specific antibodies are detected by counting gamma-emission from the pellet's bound (125)I-GAD65.(Walikonis JE, Lennon VA: Radioimmunoassay for glutamic acid decarboxylase [GAD65] autoantibodies as a diagnostic aid for stiff-man syndrome and a correlate of susceptibility to type 1 diabetes mellitus. Mayo Clin Proc 1998 December;73[12]:1161-1166)

**Western Blot (WB):** Neuronal antigens extracted aqueously from adult rat cerebellum, full-length recombinant human collapsin response-mediator protein-5 (CRMP-5), or full-length recombinant human amphiphysin protein is denatured, reduced, and separated by electrophoresis on 10% polyacrylamide gel. IgG is detected autoradiographically by enhanced chemiluminescence.(Yu Z, Kryzer TJ, Griesmann GE, et al: CRMP-5 neuronal autoantibody: marker of lung cancer and thymoma-related autoimmunity. Ann Neurol 2001 February;49[2]:146-154)

**Cell Binding Assay (CBA):** Patient specimen is applied to a composite slide containing transfected and nontransfected HEK-293 cells. After incubation and washing, fluorescein-conjugated goat-antihuman IgG is applied to detect the presence of patient IgG binding.(Package insert: EUROIMMUN AG. Stocker W. et al: Differenzierte Autoantikorper-Diagnostik mit BIOCHIP-Mosaiken. U Conrad, K. [Hrsg] Autoantikorper. Pabst-Verlag [1998] 78-99)

**SERUM**

**Indirect Immunofluorescence Assay (IFA):** Before testing, patient's specimen is preabsorbed with liver powder to remove nonorgan-specific autoantibodies. After applying to a composite substrate of frozen mouse tissues (brain, kidney, and gut) and washing, fluorescein-conjugated goat-antihuman IgG is applied to detect the distribution and pattern of patient IgG binding.(Pittock SJ, Kryzer TJ, Lennon VA: Paraneoplastic antibodies coexist and predict cancer, not neurological syndrome. Ann Neurol 2004;56:715-719)

**Western Blot (WB): N**euronal antigens extracted aqueously from adult rat cerebellum, full-length recombinant human collapsin response-mediator protein-5 (CRMP-5), or full-length recombinant human amphiphysin protein is denatured, reduced, and separated by electrophoresis on 10% polyacrylamide gel. IgG is detected autoradiographically by enhanced chemiluminescence.(Yu Z, Kryzer TJ, Griesmann GE, et al: CRMP-5 neuronal autoantibody: marker of lung cancer and thymoma-related autoimmunity. Ann Neurol 2001 February;49[2]:146-154)

**Cell-Binding Assay (CBA):** Patient specimen is applied to a composite slide containing transfected and nontransfected HEK-293 cells. After incubation and washing, fluorescein-conjugated goat-antihuman IgG is applied to detect the presence of patient IgG binding.( Package insert: IIFT: Neurology Mosaics, Instructions for the indirect immunofluorescence test. EUROIMMUN, Lubeck, Germany, FA_112d-1_A_UK_C13, 02/25/2019)

**Radioimmunoassay (RIA):** Duplicate aliquots of patient specimen are incubated with I(125)-labeled antigen. Immune complexes, formed by adding secondary (goat)-antihuman immunoglobulin, are pelleted by centrifugation and washed. Gamma emission from the washed pellet is counted, and mean counts per minute (cpm) are compared with results yielded by high-positive and -negative control sera. Specimen yielding cpm higher than the background cpm yielded by normal human specimen are retested to confirm positivity and titrated as necessary to obtain a value in the linear range of the assay. The antigen binding capacity (nmol per liter) is calculated from the cpm precipitated at a dilution yielding a linear range value.(Griesmann GE, Kryzer TJ, Lennon VA: Autoantibody profiles of myasthenia gravis and Lambert-Eaton myasthenic syndrome. In Manual of Clinical and Laboratory Immunology. Sixth edition. Edited by NR Rose, RG Hamilton, et al. Washington, DC, ASM Press, 2002, pp 1005-1012)

**Live-cell Assay (LCA):** Muscle acetylcholine receptor (AChR) modulating antibodies are detected by incubating the patient's serum for 14 hours with viable, noninnervated, monolayer cultures of human muscle cells. Percent loss of surface AChR is quantitated by probing with (125)I-alpha-bungarotoxin.(Howard FM Jr, Lennon VA, Finley J, et al: Clinical correlations of antibodies that bind, block, or modulate human acetylcholine receptors in myasthenia gravis. Ann NY Acad Sci 1987;505:526-538)
